# Recent progress towards development of effective systemic chemotherapy for the treatment of malignant brain tumors

**DOI:** 10.1186/1479-5876-7-77

**Published:** 2009-09-01

**Authors:** Hemant Sarin

**Affiliations:** 1 National Institute of Biomedical Imaging and Bioengineering, National Institutes of Health, Bethesda, Maryland, USA

## Abstract

Systemic chemotherapy has been relatively ineffective in the treatment of malignant brain tumors even though systemic chemotherapy drugs are small molecules that can readily extravasate across the porous blood-brain tumor barrier of malignant brain tumor microvasculature. Small molecule systemic chemotherapy drugs maintain peak blood concentrations for only minutes, and therefore, do not accumulate to therapeutic concentrations within individual brain tumor cells. The physiologic upper limit of pore size in the blood-brain tumor barrier of malignant brain tumor microvasculature is approximately 12 nanometers. Spherical nanoparticles ranging between 7 nm and 10 nm in diameter maintain peak blood concentrations for several hours and are sufficiently smaller than the 12 nm physiologic upper limit of pore size in the blood-brain tumor barrier to accumulate to therapeutic concentrations within individual brain tumor cells. Therefore, nanoparticles bearing chemotherapy that are within the 7 to 10 nm size range can be used to deliver therapeutic concentrations of small molecule chemotherapy drugs across the blood-brain tumor barrier into individual brain tumor cells. The initial therapeutic efficacy of the Gd-G5-doxorubicin dendrimer, an imageable nanoparticle bearing chemotherapy within the 7 to 10 nm size range, has been demonstrated in the orthotopic RG-2 rodent malignant glioma model. Herein I discuss this novel strategy to improve the effectiveness of systemic chemotherapy for the treatment of malignant brain tumors and the therapeutic implications thereof.

## Background

Malignant brain tumors consist of high-grade primary brain tumors such as malignant gliomas[1], and metastatic lesions to the brain from peripheral cancers such as lung, breast, renal, gastrointestinal tract, and melanoma[2,3]. Glioblastoma, the highest grade of malignant glioma, is the most common high-grade primary brain tumor in adults[4,5]. Overall, metastatic brain tumors are the most common brain tumors in adults, as 10% to 20% of patients with a malignant peripheral tumor develop brain metastases[2,3,6]. Even though malignant gliomas are generally treated with a combination of surgery, radiotherapy and systemic chemotherapy[7,8], and metastatic brain tumors with a combination of surgery and radiotherapy [9-11], the overall long-term prognosis of patients with these tumors, whether primary or metastatic, remains poor. Patient median survival times typically range between 3 and 16 months [12-16], and the percentage of patients alive at 5 years ranges between 3% and 10%[12,13,16,17]. In the treatment of both malignant gliomas and metastatic brain tumors, surgery and radiotherapy are more effective when used in combination[7-11,18-20]. In the treatment of malignant gliomas, there some minimal additional benefit of systemic chemotherapy[8,15,20-27]; and in the treatment of metastatic brain tumors, it remains unclear as to if there is any additional benefit of systemic chemotherapy[9,10,28-31].

Systemic chemotherapy consists of small molecule chemotherapy drugs[8,32] that are drugs of molecular weights (MW) less than 1 kDa and diameters less than 1 to 2 nm. These small molecule chemotherapy drugs include traditional drugs that target the cell cycle, for example, DNA alkylating drugs, and newer investigational drugs that target cell surface receptors and associated pathways, for example, tyrosine kinase inhibitors[8,32]. The ineffectiveness of these chemotherapy drugs in treating malignant brain tumors has been attributed to the blood-brain barrier (BBB) being a significant impediment to the transvascular extravasation of drug fraction across the barrier into the extravascular compartment of tumor tissue[29,33-35]. However, the pathologic BBB of malignant brain tumor microvasculature, also known as the blood-brain tumor barrier (BBTB), is porous[36,37]. Contrast enhancement of malignant brain tumors on MRI is due to the transvascular extravasation of Gd-DTPA (Magnevist, MW 0.938 kDa) across the pores in the BBTB into the extravascular extracellular compartment of tumor tissue[38,39].

## Historical strategies to improve the effectiveness of systemic chemotherapy

Historically, two different strategies have been employed in the effort to improve the effectiveness of small molecule systemic chemotherapy in treating malignant brain tumors, although neither strategy has been particularly effective. The first strategy has been to elevate small molecule drug concentrations within the extravascular extracellular compartment of tumor tissue. One approach to this strategy has been the use of lipophilic small molecule drugs for increased permeation of drug fraction across endothelial cells of the BBTB[40,41]. The effectiveness of this approach has been limited due to drug binding to plasma proteins[42], in addition to the efflux of a significant proportion of extravasated drug fraction back into systemic circulation by BBTB multi-drug resistance pumps such as p-glycoprotein[35,43]. Other approaches to this strategy include the administration of drugs intra-arterially to maximize first-pass drug delivery across the BBTB [44-46], and the temporary opening of the junctions between endothelial cells of the BBTB to enhance the permeation of drugs across the BBTB[34,47,48]. The overall ineffectiveness of these approaches can be attributed to the fact that there is only a transient elevation in drug concentrations within extravascular extracellular compartment of tumor tissue due to the short blood half-life of small molecule chemotherapy [49-55], which precludes the accumulation of drug fraction to therapeutic concentrations within individual brain tumor cells.

The second strategy has been to increase the blood half-life of small molecule chemotherapy. One approach to this strategy has been the intravenous co-administration of labradimil (RMP-7, Cereport), a metabolically stable bradykinin B2 receptor agonist, during the intravenous administration of small molecule chemotherapy drugs such as carboplatin. Although the co-administration of labradimil increases the blood half-life of small molecule chemotherapy drugs [56-59], the increase in drug blood half-life is temporary[60], which again, precludes the accumulation of drug fraction to therapeutic concentrations within individual brain tumor cells. Another approach to this strategy has been the use of continuous chemotherapy dosing schemes[61,62]. The potential effectiveness of this approach, however, has been limited by the systemic toxicity associated with it, which is due to the non-specific accumulation of small molecule drugs within normal tissues, as these drugs are small enough to permeate across endothelial barriers of normal tissue microvasculature [61-64].

In more recent years, slow sustained-drug release formulations of small molecule chemotherapy drugs have been developed by the non-covalent attachment of chemotherapy drugs to polymers or the encapsulation of drugs within liposomes[65,66]. Such nanoparticle-based drug release formulations are intravascular free drug reservoirs with long blood half-lives, since these spherical nanoparticles generally range between 30 nm and 200 nm in diameter [67-69], and are significantly larger than the physiologic upper limit of pore size in the BBTB of malignant brain tumor microvasculature. Since nanoparticle-based drug release formulations remain intravascular within brain tumor microvasculature, free drug is slowly released into systemic circulation, and not directly within individual brain tumor cells. Therefore, nanoparticle-based slow sustained-drug release formulations of small molecule chemotherapy drugs that are larger than the 12 nm physiologic upper limit of pore size in the BBTB result in sub-therapeutic drug concentrations within individual brain tumor cells, since free drug is not released directly within individual brain tumor cells [70-72].

## Novel strategy to improve the effectiveness of systemic chemotherapy

The novel strategy that I propose here to improve the effectiveness of systemic chemotherapy in the treatment of malignant brain tumors is based on my two recent observations[59,73,74]. The first observation being that spherical nanoparticles smaller than 12 nm in diameter, but not larger, can extravasate across the porous BBTB of malignant brain tumor microvasculature[73,74]. The second observation being that the subset of nanoparticles ranging between 7 nm and 10 nm in diameter are of sizes sufficiently smaller than the 12 nm physiologic upper limit of pore size within the BBTB and maintain peak blood concentrations for several hours, and therefore, can accumulate over time to effective concentrations within individual brain tumor cells[73,74]. Based on these two observations, spherical nanoparticles ranging between 7 nm and 10 nm in diameter can be used to deliver therapeutic concentrations of small molecule chemotherapy drugs across the BBTB and into individual malignant brain tumor cells. Since systemically administered nanoparticles within this 7 to 10 nm size range would not extravasate across the normal BBB of brain microvasculature [73-77] or across the endothelial barriers of most normal tissue microvasculature[59,63,78,79], these nanoparticles would extravasate "selectively" across the porous BBTB of malignant brain tumor microvasculature.

We have recently demonstrated that an imageable nanoparticle bearing chemotherapy within the 7 to 10 nm size range at delivers therapeutic concentrations of small molecule chemotherapy across the BBTB into individual brain tumor cells. This prototype of an imageable nanoparticle bearing small molecule chemotherapy is a gadolinium (Gd)-diethyltriaminepentaacetic acid (DTPA) chelated generation 5 (G5) polyamidoamine (PAMAM) dendrimer with a proportion of the available terminal amines conjugated via pH-sensitive covalent linkages to doxorubicin (Adriamycin; MW 0.580 kDa), a fluorescent small molecule chemotherapy drug that intercalates with DNA and inhibits the DNA replication process. The initial therapeutic efficacy of the Gd-G5-doxorubicin dendrimer has been tested in the orthotopic RG-2 rodent malignant glioma model. In this rodent glioma model we have found that one dose of the Gd-G5-doxorubicin dendrimer is significantly more effective than one dose of free doxorubicin at inhibiting the growth of RG-2 gliomas for approximately 24 hours.

## The physiologic upper limit of pore size in the BBTB of malignant brain tumor microvasculature

Simple diffusion of nutrients and metabolites between tumor cells and pre-existent host tissue microvasculature is only sufficient to sustain solid tumor growth to a volume of 1 to 2 mm^3^[80]. Additional tumor growth requires the formation of new microvasculature, a process that is mediated by vascular endothelial growth factor (VEGF)[81]. The new tumor microvasculature induced by VEGF is discontinuous due to the presence of anatomic defects within and between endothelial cells of the tumor barrier[82,83]. These anatomic defects in the tumor barrier can be several hundred nanometers wide [84-86]. For this reason, the endothelial barrier of malignant solid tumor microvasculature is more permeable to the transvascular passage of macromolecules than the endothelial barriers of normal tissue microvasculature including that of the kidney glomeruli[83,87]. Even though the anatomic defects within the endothelial barriers of malignant solid tumor microvasculature are relatively wide [84-86], we have found that in the physiologic state *in vivo *there is a fairly well-defined upper limit of pore size, which is approximately 12 nm, independent of whether the location of the malignant solid tumor is within the brain and the central nervous system[73,74], or outside of it, in peripheral tissues[74].

**Figure 1:**
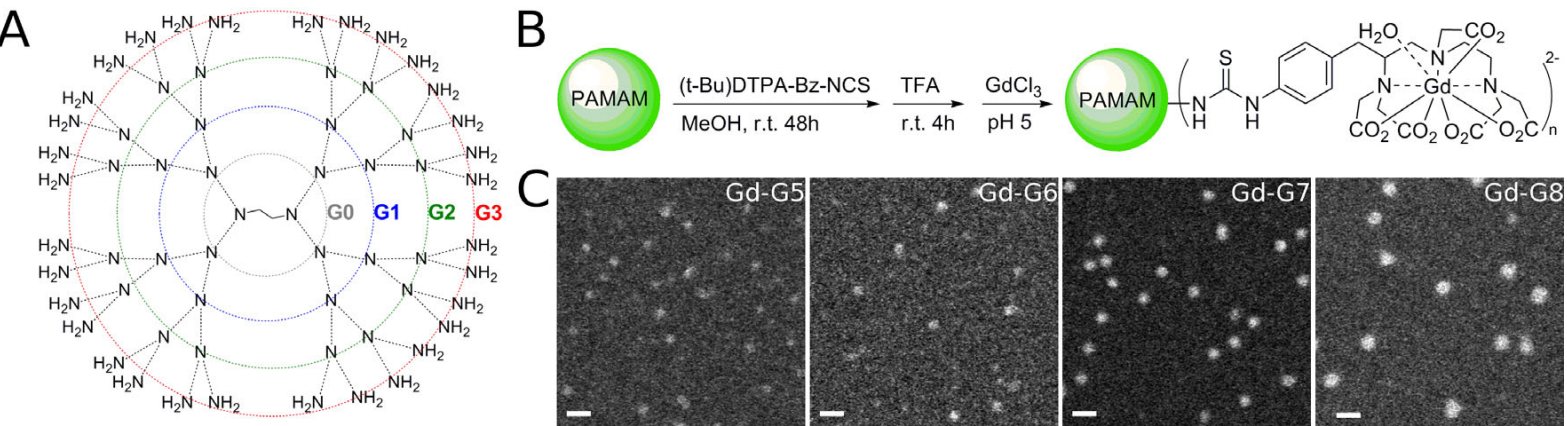
**Synthesis of gadolinium (Gd)-diethyltriaminepentaacetic acid (DTPA) conjugated polyamidoamine (PAMAM) dendrimers and images of higher generation (G) Gd-dendrimers with annular dark-field scanning transmission electron microscopy**. A) Illustrations of naked PAMAM dendrimer generations from the ethylenediamine core (G0) to generation 3 (G3). The exterior of naked PAMAM dendrimers is positively charged due to the presence of terminal amine groups. The number of terminal amine groups doubles with each successive generation. B) Synthetic scheme for the production of Gd-DTPA conjugated PAMAM dendrimers. The conjugation of Gd-DTPA (charge -2) to the terminal amine groups neutralizes the positive charge on the dendrimer exterior. C) Annular dark-field scanning transmission electron microscopy images of Gd-G5, Gd-G6, Gd-G7, and Gd-G8 dendrimers adsorbed onto an ultrathin carbon support film. The average diameter of sixty Gd-G7 dendrimers is 11.0 ± 0.7 nm and that of sixty Gd-G8 dendrimers is 13.3 ± 1.4 nm (mean ± standard deviation). Scale bar = 20 nm. Adapted from reference[73].

Polyamidoamine (PAMAM) dendrimers functionalized with gadolinium (Gd)-diethyltriaminepentaacetic acid (DTPA), a small molecule MRI contrast agent, range in diameter between 1.5 nm (Gd-DTPA PAMAM dendrimer generation 1, Gd-G1) and 14 nm (Gd-DTPA PAMAM dendrimer generation 8, Gd-G8)[73,74]. Since each Gd-DTPA moiety carries a charge of -2, conjugation of Gd-DTPA to a significant proportion of the terminal amine groups on PAMAM dendrimer exterior neutralizes the positively charged exterior of naked PAMAM dendrimers (Figure 1, panels A and B). The masses of Gd-G5 through Gd-G8 dendrimer particles are sufficient enough for particle visualization by annular dark-field scanning transmission electron microscopy (ADF STEM)[73,74,88], and the sizes of Gd-G7 and Gd-G8 dendrimer particles are large enough for estimation of particle diameters, which are approximately 11 nm for Gd-G7 dendrimers and approximately 13 nm for Gd-G8 dendrimers (Figure 1, panel C)[73,74].

Particle transvascular extravasation across the BBTB and accumulation within the extravascular compartment of brain tumor tissue has been historically measured with quantitative autoradiography [89-91], which only provides information about particle accumulation once per specimen at post-mortem, or by intravital fluorescence microscopy[92], which requires that tumors be grown in dorsal window chambers and provides low-resolution real-time data. In more recent years, dynamic contrast-enhanced MRI has been used to visualize the degree of particle transvascular extravasation across the BBTB[59,73,93,94], since it is non-invasive and provides high-resolution real-time data. With dynamic contrast-enhanced MRI it is possible to measure over time the degree of Gd-dendrimer extravasation across the BBTB and accumulation in the extravascular compartment of tumor tissue. The Gd-dendrimer concentration in tumor tissue can be estimated by the *in vivo *measurement of tumor tissue MRI signal at baseline (*T*_10_) and then again following the intravenous infusion of the Gd-dendrimer (*T*_1_), and the *in vitro *measurement of the molar relaxivity (*r*_1_) of the Gd-dendrimer, which is the proportionality constant for conversion of Gd signal to Gd concentration[73,74,95].

**Figure 2:**
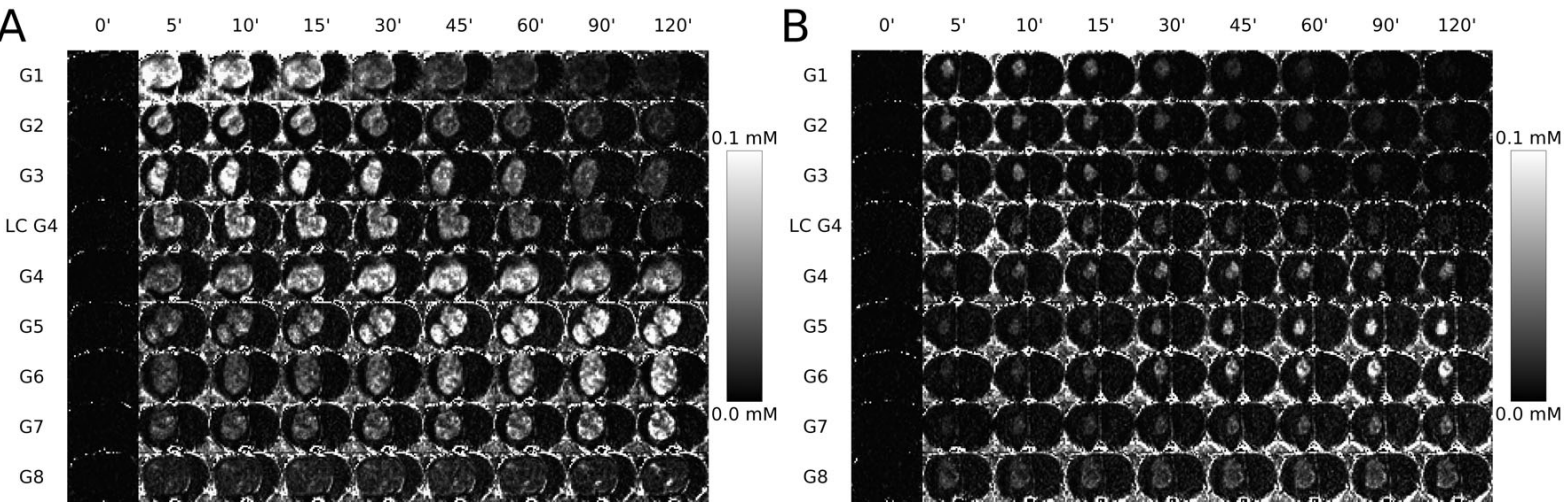
**Dynamic contrast-enhanced MRI-based Gd concentration maps of Gd-dendrimer distribution within large and small RG-2 rodent gliomas over time**. A) Large RG-2 gliomas. Gd-G1 thorough Gd-G7 dendrimers extravasate across the BBTB of the microvasculature of large RG-2 gliomas. After extravasating across the BBTB, Gd-G1 through Gd-G4 dendrimers only remain temporarily within the extravascular compartment of tumor tissue, as these lower Gd-dendrimer generations maintain peak blood concentrations for only a few minutes. The Gd-G5 through Gd-G7 dendrimers accumulate over time within the extravascular compartment of tumor tissue, as these generations maintain peak blood concentrations for several hours. The Gd-G8 dendrimers remain intravascular, since Gd-G8 dendrimers are larger than the physiologic upper limit of pore size in the BBTB of large RG-2 gliomas. RG-2 glioma volumes (mm^3^): Gd-G1, 104; Gd-G2, 94; Gd-G3, 94; lowly conjugated (LC) Gd-G4, 162; Gd-G4, 200; Gd-G5, 230; Gd-G6, 201; Gd-G7, 170; Gd-G8, 289. B) Small RG-2 gliomas. Gd-G1 thorough Gd-G6 dendrimers extravasate across the BBTB of the microvasculature of small RG-2 gliomas. Since small RG-2 gliomas are less vascular than large RG-2 gliomas, there is a relative lack of accumulation of the lower Gd-dendrimer generations in the extravascular compartment of small RG-2 gliomas as compared to large RG-2 gliomas (panel A). This is especially evident in the case of Gd-G1 dendrimers, which maintain peak blood concentrations for the shortest time period of all the Gd-dendrimer generations. Gd-G5 and Gd-G6 dendrimers accumulate over time within the extravascular compartment of even the small RG-2 gliomas, since these generations maintain peak blood concentrations fro several hours and are smaller than the physiologic upper limit of pore size in the BBTB. Both Gd-G7 and Gd-G8 dendrimers remain intravascular in small RG-2 gliomas, since both Gd-G7 and Gd-G8 dendrimers are larger than the physiologic upper limit of pore size in the BBTB of small RG-2 gliomas. RG-2 glioma volumes (mm^3^): Gd-G1, 27; Gd-G2, 28; Gd-G3, 19; LC Gd-G4, 24; Gd-G4, 17; Gd-G5, 18; Gd-G6, 22; Gd-G7, 24; Gd-G8, 107. Respective Gd-dendrimer generations administered intravenously over 1 minute at a Gd dose of 0.09 mmol Gd/kg animal body weight. Scale ranges from 0 mM [Gd] to 0.1 mM [Gd]. Adapted from reference[73].

We have determined that Gd-G1 through Gd-G7 dendrimer particles traverse the pores of the BBTB of RG-2 rodent malignant glioma microvasculature and enter the extravascular compartment of tumor tissue, but that the Gd-G8 dendrimer particles remain intravascular (Figure 2, panels A and B)[73,74]. Therefore, the physiologic upper limit of pore size within the BBTB of malignant brain tumor microvasculature is approximately 12 nm, since Gd-G7 dendrimers, being approximately 11 nm in diameter, can extravasate across the BBTB, whereas Gd-G8 dendrimers, being approximately 13 nm in diameter, cannot[73,74]. On comparison of the physiologic upper limit of pore size in the BBTB of small RG-2 glioma microvasculature to that of the BBTB of large RG-2 glioma microvasculature, we have found that Gd-G1 through Gd-G6 dendrimers also readily traverse pores within the BBTB of small RG-2 glioma microvasculature (Figure 2, panel B)[73]. However, Gd-G7 dendrimers do not readily extravasate across the BBTB of small RG-2 glioma microvasculature (Figure 2, panel B)[73]. This finding is consistent with the likelihood that the physiologic upper limit of pore size in the BBTB of the microvasculature of early, less mature and smaller malignant brain tumor colonies is 1 to 2 nanometers lower than that of the BBTB of the microvasculature of late, more mature and larger malignant brain tumors. Since most small molecule chemotherapy drugs are less than 1 to 2 nm in diameter, a slightly lower physiologic upper limit of pore size in the BBTB of the microvasculature of early, less mature and smaller malignant brain tumor colonies does not explain why small molecule chemotherapy drugs do not accumulate to effective concentrations within the extravascular compartment of early, less mature and smaller malignant brain tumor colonies, whether primary or metastatic.

## Significance of the luminal glycocalyx layer of the BBTB of malignant brain tumor microvasculature

The well-defined physiologic upper limit of pore size in the BBTB of 12 nm would be attributable to the presence of a luminal glycocalyx layer overlaying the anatomic defects within the BBTB. Since the fibrous matrix of the glycocalyx overlaying endothelial barriers may be several hundred nanometers thick [96-100], it would be the "nanofilter" that serves as the main point of resistance to the transvascular passage of spherical particles larger than 12 nm in diameter across the BBTB. Therefore, in the physiologic state *in vivo*, the presence of the glycocalyx would render the underlying endothelial cells of the BBTB inaccessible to the transvascular passage of liposomes, viruses, bacteria, or cells, unless the glycocalyx was stretched, degraded, or disrupted in some manner [101-107]. Furthermore, the glycocalyx layer would also be expected to offer considerable resistance to the transvascular passage of non-spherical particles with sizes at the cusp of the physiologic upper limit of pore size including monoclonal antibodies (immunoglobulin G, IgG), which have sizes of approximately 11 nm based on the calculation of antibody diffusion coefficients in viscous fluids[108]. The 12 nm physiologic upper limit of pore size is the likely reason why monoclonal antibody-based systemic chemotherapy has not been effective at treating malignant solid tumors[109].

## Nanoparticle blood half-life and particle accumulation within individual brain tumor cells

With dynamic-contrast enhanced MRI we have characterized the relationship between Gd-dendrimer blood half-life and transvascular extravasation across the BBTB of RG-2 rodent malignant gliomas. Based on our findings, it is evident that spherical nanoparticles ranging between 7 nm an 10 nm in diameter maintain peak blood concentrations for several hours and are sufficiently smaller than the 12 nm physiologic upper limit of pore size in the BBTB to accumulate to effective concentrations within individual brain tumor cells[73,74]. For spherical particles that are smaller than 6 nm in diameter, the distribution of particles within the extravascular compartment of tumor tissue becomes more focal as particle size increases, since these particles maintain peak blood concentrations for only minutes[73]. However, for spherical particles that range between 7 nm and 10 nm in diameter, the distribution of particles within the extravascular compartment of tumor tissue is widespread, irrespective of particle size, since these particles maintain peak blood concentrations for several hours[73,74].

**Figure 3:**
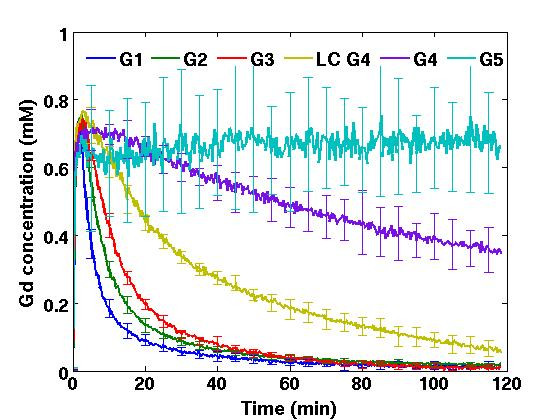
**Steady-state blood concentrations of successively higher generation Gd-dendrimers over time in rodents**. Gd-G1 dendrimers (MW 6 kDa), Gd-G2 dendrimers (MW 11 kDa), Gd-G3 dendrimers (MW 19 kDa), lowly conjugated (LC) Gd-G4 dendrimers (MW 25 kDa), and standard Gd-G4 dendrimers (MW 40 kDa) maintain peak blood concentrations for only a few minutes. Gd-G5 dendrimers (MW 80 kDa) maintain peak blood concentrations for over 2 hours. Gd-G6 dendrimers (MW 130 kDa), Gd-G7 dendrimers (MW 330 kDa), and Gd-G8 dendrimers (MW 597 kDa) also maintain peak blood concentrations for over 2 hours similar to those of Gd-G5 dendrimers (concentration profiles not shown for purposes of figure clarity). Respective Gd-dendrimer generations administered intravenously over 1 minute at a Gd dose of 0.09 mmol Gd/kg animal body weight. Blood concentrations of Gd-dendrimers over time measured in the superior sagittal sinus. Gd-G1 (n = 4), Gd-G2 (n = 6), Gd-G3 (n = 6), lowly conjugated (LC) Gd-G4 (n = 4), Gd-G4 (n = 6), Gd-G5 (n = 6), Gd-G6 (n = 5), Gd-G7 (n = 5), and Gd-G8 (n = 6). Error bars represent standard deviations. Adapted from reference[73].

Spherical particles smaller than 6 nm in diameter (MW less than 40 to 50 kDa)[88,110-112], which is the size range of Gd-G1 through Gd-G4 dendrimers, possess relatively short blood half-lives[73], and therefore, maintain peak blood concentrations for only minutes (Figure 3)[73], as these particles are small enough to be efficiently filtered by the kidney glomeruli[113]. As such, particles smaller than 6 nm only remain temporarily within the extravascular compartment of tumor tissue (Figure 2, rows 1 through 5)[73], which would not be sufficient time for particles to accumulate to therapeutic concentrations within individual brain tumor cells. The blood half-life of small molecule chemotherapy drugs would be even shorter than that of the smallest Gd-dendrimer, the Gd-G1 dendrimer (Figure 2, row 1)[73]. Therefore, the short blood half-life of small molecule chemotherapy drugs would be the primary reason why these small drugs do not accumulate to therapeutic concentrations within individual brain tumor cells after extravasating across the porous BBTB of malignant brain tumor microvasculature.

**Figure 4:**
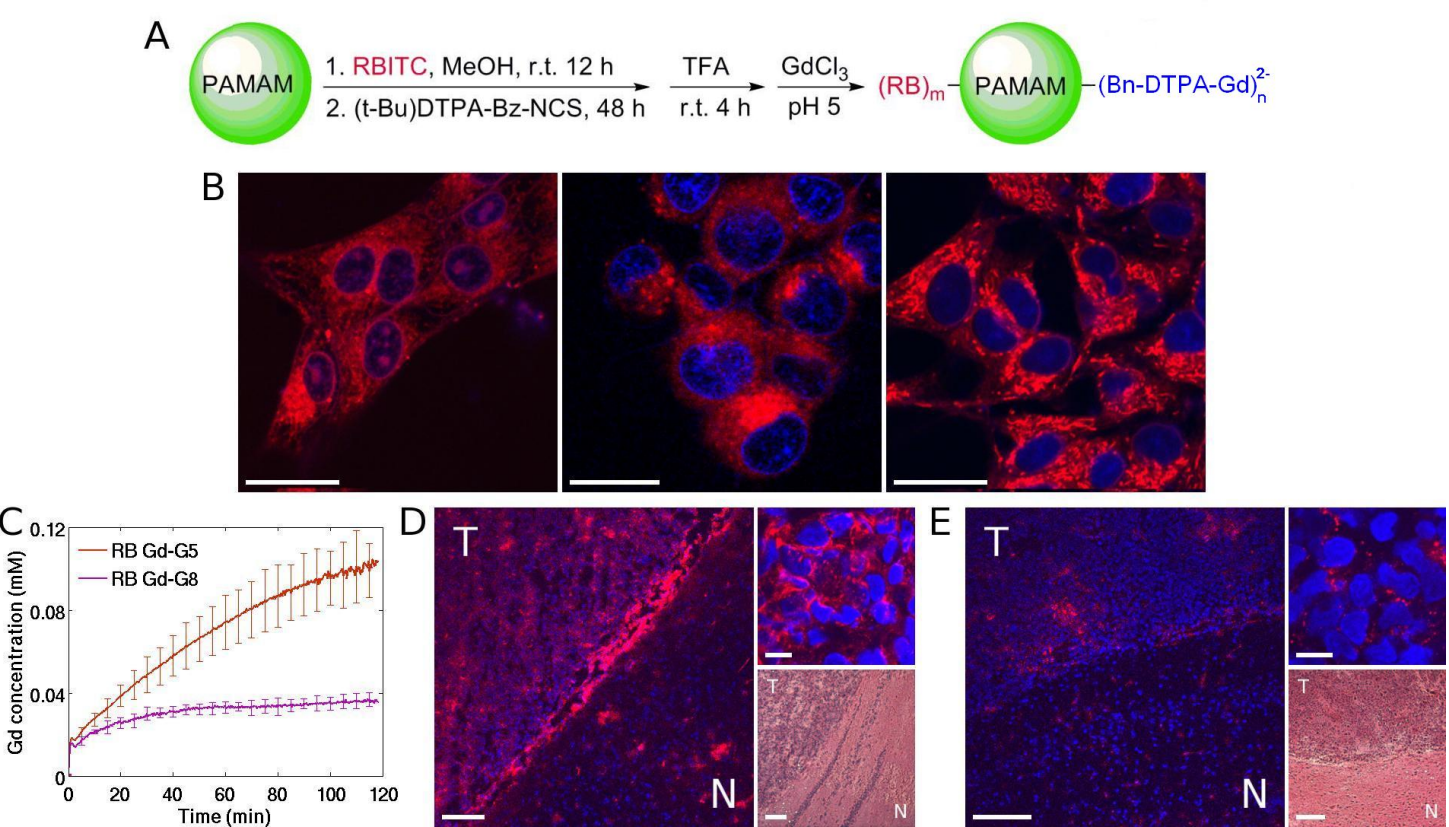
**Synthesis of rhodamine B dye (RB) conjugated Gd-dendrimers and fluorescence microscopy of rhodamine B conjugated Gd-dendrimer uptake in cultured RG-2 glioma cells versus in RG-2 glioma cells of harvested RG-2 glioma tumor specimens**. A) Synthetic scheme for production of rhodamine B dye conjugated Gd-dendrimers. Rhodamine B and DTPA are conjugated to the naked dendrimer terminal amines via stable covalent bonds. In functionalized dendrimers, approximately 35% of the terminal amines are occupied by Gd-DTPA, and approximately 7% of the terminal amines are occupied by rhodamine B. B) *In vitro *fluorescence microscopy of cultured RG-2 glioma cells incubated for 4 hours in media containing either rhodamine B conjugated Gd-G2 dendrimers (left), rhodamine B conjugated Gd-G5 dendrimers (middle), or rhodamine B conjugated Gd-G8 dendrimers (right) at a concentration of 7.2 μM with respect to rhodamine B. Scale bars = 20 μm. Rhodamine B conjugated Gd-G2 dendrimers enter RG-2 glioma cells, and in some cases, the cell nuclei (left). Rhodamine B conjugated Gd-G5 dendrimers (middle) and rhodamine B conjugated Gd-G8 dendrimers (right) enter the cytoplasm of RG-2 glioma cells, but do not localize within the nuclei. C) Dynamic contrast-enhanced MRI-based Gd concentration curves of RG-2 glioma tumor tissue over time following the intravenous bolus of 0.06 mmol Gd/kg of rhodamine B conjugated Gd-G5 dendrimers (n = 6) and rhodamine B conjugated Gd-G8 dendrimers (n = 2). There is substantial extravasation of rhodamine B conjugated Gd-G5 dendrimers across the BBTB, which is more pronounced than that of Gd-G5 dendrimers across the BBTB. There is also some extravasation of rhodamine B conjugated Gd-G8 dendrimers across the BBTB, which is not the case for Gd-G8 dendrimers. D) *Ex vivo *low power fluorescence microscopy of RG-2 glioma tumor and surrounding brain tissue harvested at 2 hours following the intravenous bolus of rhodamine B conjugated Gd-G5 dendrimers. There is substantial accumulation of rhodamine B conjugated Gd-G5 dendrimers within tumor tissue, and some in surrounding normal brain tissue (left, T = tumor, N = normal, scale bar = 100 μm). High power image of RG-glioma tumor shows subcellular localization of rhodamine B conjugated Gd-G5 dendrimers within individual RG-2 malignant glioma cells (upper right, scale bar = 20 μm). H&E stain of tumor and surrounding brain (lower right, scale bar = 100 μm). Tumor volume is 31 mm^3^. E) *Ex vivo *low power fluorescence microscopy of RG-2 glioma tumor and surrounding brain tissue harvested at 2 hours following the intravenous bolus of rhodamine B conjugated Gd-G8 dendrimers. There is some minimal accumulation of rhodamine B conjugated Gd-G8 dendrimers within brain tumor tissue (left, T = tumor, N = normal, scale bar = 100 μm). High power confirms there is some minimal subcellular localization of rhodamine B conjugated Gd-G8 dendrimers within individual RG-2 glioma cells (upper right, scale bar = 20 μm). H&E stain of tumor and surrounding brain (lower right, scale bar = 100 μm). Tumor volume is 30 mm^3^. Rhodamine B conjugated Gd-G5 dendrimers and rhodamine B conjugated Gd-G8 dendrimers administered intravenously over 1 minute at a Gd dose of 0.06 mmol Gd/kg animal body weight. Adapted from reference[73].

Spherical particles greater than 7 nm in diameter (MW greater than 70 to 80 kDa)[88,110-112], which is the size range of Gd-G5 through Gd-G8 dendrimers, possess relatively long particle blood half-lives[74], and therefore, maintain peak blood concentrations for several hours (Figure 3)[73,74], as these particles are too large to be filtered by the kidney glomeruli. Particles ranging between 7 nm and 10 nm in diameter, those being Gd-G5 and Gd-G6 dendrimers, slowly accumulate over 2 hours within the extravascular compartment of even small RG-2 malignant gliomas (Figure 2, rows 6 and 7)[73]. Due to the prolonged residence time of particles within the extravascular compartment of tumor tissue, there is significant endocytosis of particles into individual RG-2 glioma cells, which is evident on fluorescence microscopy of tumor tissue harvested 2 hours following the intravenous administration of rhodamine B dye conjugated Gd-G5 dendrimers (Figure 4, panel D)[73]. This finding indicates that spherical nanoparticles ranging between 7 nm and 10 nm in diameter can be used to deliver therapeutic concentrations of small molecule chemotherapy drugs across the BBTB and into individual malignant glioma cells. Furthermore, with spherical particles in the 7 to 10 nm size range, it would be possible to deliver therapeutic concentrations of small molecule chemotherapy drugs across the BBTB of the microvasculature of early, less mature and smaller brain tumor colonies (Figure 2, panel B, rows 6 and 7), even though these smaller tumors are less vascular than late, more mature and larger malignant brain tumors[59,73,90,91,114,115].

## Issue of positive charge on the nanoparticle exterior

Small molecules and peptides with significant focal positive charges[116,117] can disrupt the luminal glycocalyx layer, which is a polysaccharide matrix bearing an overall negative charge[96]. When positively charged small molecules are attached to the exterior of nanoparticles with long blood half-lives, the prolonged exposure of the cationic particle surface to the glycocalyx can result in its significant disruption[116,118]. Prior to our recent studies on the physiologic upper limit of the pore size within the BBTB of malignant brain tumors and the blood-tumor barrier (BTB) of malignant peripheral tumors[73,74], the pore size within the BBTB and BTB had been probed by intravital fluorescence microscopy 24 hours following the intravenous infusion of cationic liposomes and microspheres labeled on the exterior with rhodamine B dye[116,119,120]. Since, in these prior studies, the intravital fluorescence microscopy of particle extravasation across the BBTB and BTB was performed 24 hours following the intravenous infusion of cationic nanoparticles[119,120], it is to be expected that the measured physiologic pore sizes with this approach would approximate the sizes of anatomic defects underlying the glycocalyx[85], as 24 hours would be sufficient time for cationic nanoparticles to completely disrupt the glycocalyx and expose the underlying anatomic defects within the respective tumor barriers.

The positive charge on exterior of the naked PAMAM dendrimer generations is neutralized by the conjugation of Gd-DTPA (charge -2) to a significant proportion of the terminal amines. Therefore, intravenously administered Gd-DTPA conjugated dendrimer generations do not disrupt the glycocalyx overlaying the already porous BBTB and the normally non-porous BBB. However, when rhodamine B dye is conjugated to Gd-dendrimer terminal amines this positively charged molecule protrudes above the negatively charged Gd-DTPA moieties and re-introduces positive charge to the particle exterior, which results in positive charge-induced disruption of the glycocalyx of the already porous BBTB and the normally non-porous BBB. The disruption of the glycocalyx overlaying the already porous BBTB results in enhanced extravasation of rhodamine B conjugated Gd-G5 dendrimers across the BBTB and in some minimal extravasation of rhodamine B conjugated Gd-G8 dendrimers across the BBTB, which is evident *in vivo *on dynamic contrast-enhanced MRI 5 to 10 minutes following the intravenous infusion of the respective rhodamine B conjugated Gd-dendrimer generations(Figure 4, panel C)[73]. It is also evident *ex vivo *on fluorescence microscopy of RG-2 glioma specimens harvested at 2 hours following intravenous infusion of the respective rhodamine B conjugated Gd-dendrimer generations (Figure 4, panels D and E)[73]. This finding is consistent with the greater exposure of underlying pre-existent anatomic defects in the BBTB and a slight increase in the physiologic upper limit of pore size in the BBTB due to positive charge-induced toxicity to the glycocalyx.

The disruption of the glycocalyx overlaying the normally non-porous BBB results in some non-selective minimal extravasation of both rhodamine B conjugated Gd-G5 and rhodamine B conjugated Gd-G8 dendrimers across the BBB, which is evident *in vivo *on dynamic contrast-enhanced MRI 30 to 45 minutes following the intravenous infusion of the respective rhodamine B conjugated Gd-dendrimer generations[73]. It is also evident *ex vivo *on fluorescence microscopy of the normal brain tissue surrounding RG-2 glioma tumor tissue (Figure 4, panels D and E)[73]. This finding is consistent with the formation of new anatomic defects within and between endothelial cells of the BBB following disruption of the overlaying glycocalyx. On the basis of our recent findings[73,74], in the context of what has been previously reported[106,107,121], it is evident that the presence of positive charge on the nanoparticle exterior enhances the transvascular extravasation of particles across pathologic tumor barriers, and also across normal endothelial barriers, by positive charge-induced toxicity to the luminal glycocalyx layer.

## The prototype of an imageable nanoparticle bearing chemotherapy within the 7 to 10 nm size range: The Gd-G5-doxorubicin dendrimer

**Figure 5:**
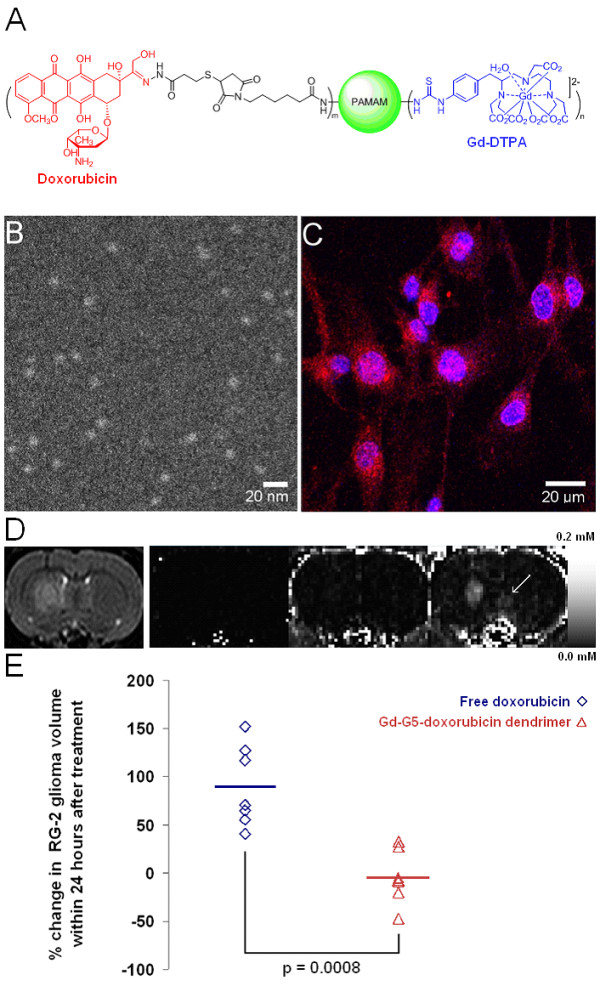
**The prototype of an imageable nanoparticle bearing chemotherapy within the 7 to 10 nm size range: The Gd-G5-doxorubicin dendrimer**. A) An illustration of the Gd-G5-doxorubicin dendrimer. Doxorubicin is conjugated to the dendrimer terminal amines by a pH-sensitive hydrazone bond, which facilitates the rapid release of doxorubicin following particle endocytosis into brain tumor cell lysosomal compartments. B) Annular dark-field scanning transmission electron microscopy image of Gd-G5-doxorubicin dendrimers. C) *In vitro *fluorescence microscopy of cultured RG-2 glioma cells incubated for 4 hours in media containing Gd-G5-doxorubicin dendrimers at a 600 nM concentration. The red fluorescence in the cytoplasm represents Gd-G5-doxorubicin dendrimers within the cytoplasm of RG-2 glioma cells. The red fluorescence within the RG-2 cell nuclei represents free doxorubicin that has been released from the Gd-G5-doxorubicn dendrimers following cleavage of the hydrazone bond, since particles larger than Gd-G2 dendrimers are too large to pass through the nuclear pores. D) *T*_2_-weighted anatomic scan image and *T*_1_-weighted dynamic contrast-enhanced MRI scan Gd concentration map images at various time points up to 60 minutes following Gd-G5-doxorubicn dendrimer infusion. The Gd-G5-doxorubicin dendrimer was administered intravenously over 2 minutes at a Gd dose of 0.09 mmol Gd/kg, which is equivalent to a doxorubicin dose of 8 mg/kg. The *T*_2_-weighted anatomic scan image shows the location of the RG-2 glioma in the right caudate of rat brain, which has a tumor volume of 16 mm^3^. The first *T*_1_-weighted dynamic contrast-enhanced MRI scan image displays the lack of contrast enhancement prior to Gd-G5 doxorubicin dendrimer infusion. The second *T*_1_-weighted dynamic contrast-enhanced MRI scan image confirms contrast enhancement in the vasculature immediately after Gd-G5-doxorubicin dendrimer infusion. The third *T*_1_-weighted dynamic contrast-enhanced MRI scan image shows that at 60 minutes following the Gd-G5-doxorubicin dendrimer infusion there is significant Gd-G5-doxorubicin accumulation within the RG-2 glioma tumor extravascular extracellular space, which confirms that the Gd-G5-doxorubicin dendrimer has extravasated slowly across the BBTB over timer due to its long blood half-life. The white arrow highlights that there is positive contrast enhancement of normal brain tissue, which indicates that there is extravasation of the Gd-G5-doxorubicin dendrimer across the normal BBB. E) Percent change in RG-2 malignant glioma volume within 24 hours. One group of orthotopic RG-2 glioma bearing animals received one intravenous 8 mg/kg dose of Gd-G5-doxorubicin dendrimer with respect to doxorubicin (n = 7), and the other group of glioma bearing animals received one 8 mg/kg dose of free doxorubicin (n = 7). Pre-treatment whole RG-2 glioma tumor volumes calculated based on initial *T*_2_-weighted anatomic scans acquired immediately prior to agent administration, and post-treatment whole RG-2 glioma tumor volumes calculated based on repeat *T*_2_-weighted anatomic scans acquired within 22 ± 2 hours for the Gd-G5-doxorubicin group and 24 ± 1 hour for the free doxorubicin group. One dose of the Gd-G5-doxorubicin dendrimer is significantly more effective than one dose of free doxorubicin at inhibiting the growth of orthotopic RG-2 malignant gliomas for approximately 24 hours. Student's two-tailed paired t-test p value < 0.0008.

Based on our finding that spherical nanoparticles ranging between 7 nm and 10 nm in diameter effectively traverse pores within the BBTB and accumulate to high concentrations within individual brain tumor cells, an imageable nanoparticle bearing chemotherapy within the 7 to 10 nm size range, the Gd-G5-doxorubicin dendrimer, has been developed (Figure 5, panel A). The Gd-G5-doxorubicin dendrimer has been visualized *in vitro *with annular dark-field scanning electron microscopy (Figure 5, panel B). Gd-DTPA was conjugated to approximately 50% of the terminal amines and doxorubicin to approximately 8% of the terminal amines of a G5 PAMAM dendrimer (Table 1), which yielded the optimal ratio of contrast agent-to-drug for dynamic contrast-enhanced MRI and systemic chemotherapy, respectively.

**Table 1:** Properties of the Gd-G5-doxorubicin dendrimer

PAMAM dendrimer generation (G)	Terminal amines (#)	Naked dendrimer molecular weight (kDa)	Gd-G5-doxorubicin dendrimer molecular weight (kDa)	Gd-DTPA conjugation (%)	Doxorubicin conjugation (%)	Molar relaxivity (mM^-1^s^-1^)
G5	128	29^#^	85^‡^	48.1	7.8	10.1^&^

# molecular weight of naked PAMAM dendrimer obtained from Dendritech, Inc.

^‡^molecular weight measured by MALDI-TOF mass spectrometry

^&^molar relaxivity of Gd-DTPA measured to be 4.1 mM^-1^s^-1^

The doxorubicin was conjugated to the Gd-G5 dendrimer terminal amines via a pH-sensitive hydrazone bond that is stable at the physiologic pH of 7.4, and labile at the acidic pH of 5.5 in lysosomal compartments [122-125]. The functionality of the pH-sensitive hydrazone bond was verified *in vitro *with fluorescence microscopy, which showed that there is accumulation of free doxorubicin in RG-2 glioma cell nuclei following the incubation of glioma cells for 4 hours in media containing Gd-G5-doxorubicin dendrimers (Figure 5, panel C). The relative stability of the hydrazone bond at physiologic pH would limit doxorubicin release in the systemic blood circulation and minimize any systemic toxicity associated with free drug release in the bloodstream, prior to particle extravasation across the BBTB. It would be expected that there would be limited free drug release within the extravascular extracellular compartment of tumor tissue after particle extravasation across the BBTB, since the extravascular extracellular compartment is significantly less acidotic than the intracellular lysosomal compartments of cells[124,126]. Furthermore, there would be rapid doxorubicin release following particle endocytosis into tumor cell lysosomal compartments, which would enable the free doxorubicin to traverse the nuclear pores and interact with the DNA. Most small molecule chemotherapy drugs act within the cell nucleus, which necessitates that free drug be released into the tumor cell cytoplasm, which would not be possible to accomplish with spherical nanoparticles larger than Gd-G2 dendrimers, as particles of sizes larger than Gd-G2 dendrimers do not appear to effectively traverse nuclear pores (Figure 4, panel B)[73].

The cytotoxicity of the Gd-G5-doxorubicin dendrimer was verified *in vitro *with RG-2 glioma cell survival measured by the MTT (3-(4,5-dimethylthiazol-2-yl)-2,5-diphenyltetrazolium bromide) assay[127]. The Gd-G5-doxorubicin dendrimer was intravenously bolused over 2 minutes to orthotopic RG-2 glioma bearing rodents at a dose of 8 mg/kg with respect to doxorubicin. On dynamic contrast-enhanced MRI over 1 hour, it was evident that the Gd-G5-doxorubicin dendrimer extravasates across the BBTB and accumulates within the extravascular compartment of brain tumor tissue over time (Figure 5, panel D). There was, however, also some transvascular extravasation of the Gd-G5-doxorubicin dendrimer across the normal BBB and non-selective accumulation of Gd-G5-doxorubicin dendrimer in normal brain tissue (Figure 5, panel D arrow), which would be attributable to the re-introduction of focal positive charge to the Gd-G5 dendrimer exterior due to the attachment of doxorubicin, which is a cationic drug[128]. Despite this drawback, one 8 mg/kg dose of Gd-G5-doxorubicin dendrimer with respect to doxorubicin was found to be significantly more effective than one 8 mg/kg dose of free doxorubicin at inhibiting the growth of orthotopic RG-2 malignant gliomas for approximately 24 hours (Figure 5, panel E). The short-term efficacy of this approach stems from the accumulation of small molecule chemotherapy to therapeutic concentrations directly within individual brain tumor cells. The long-term efficacy of this approach will need to be evaluated in various animal malignant glioma models[129,130], prior to clinical translation.

## Therapeutic implications and future perspective

The Gd-G5-doxorubicin dendrimer, being a nanoparticle bearing chemotherapy within the 7 nm to 10 nm size range, delivers therapeutic concentrations of doxorubicin across the porous BBTB of malignant brain tumors into individual tumor cells. Doxorubicin attachment to the Gd-G5-doxorubicin dendrimer via pH-sensitive hydrazone bonds facilitates rapid doxorubicin release within the brain tumor cell lysosomal compartments and the accumulation of released doxorubicin within tumor cell nuclei. The short-term efficacy of the Gd-G5-doxorubicin dendrimer in regressing RG-2 malignant gliomas stems from the effective transvascular delivery of doxorubicin across the BBTB into individual brain tumor cells. The attachment of doxorubicin to the Gd-G5 dendrimer exterior, however, re-introduces positive charge to Gd-G5-dendrimer exterior, since the positively charged doxorubicin molecules protrude above the negatively charged Gd-DTPA molecules. The presence of positive charge on the Gd-G5-doxorubicin dendrimer exterior is toxic to the luminal glycocalyx layer and results in non-selective accumulation of the Gd-G5-doxorubicin dendrimer in normal brain tissue. Therefore, in the future, cationic small molecule chemotherapy drugs will need to be conjugated by hydrazone bonds closer to the particle interior, which would minimize the re-introduction of positive charge on the particle exterior. Furthermore, in the future, it may also be advantageous to use naked half generation PAMAM dendrimers (i.e. G5.5) as substrates for conjugation of cationic molecules, since these PAMAM dendrimer generations are anionic. Other types of biocompatible dendrimers, for example, those that are amino acid-based, would also be appropriate substrates for functionalization, provided there is no net positive charge on the functionalized particle surface.

Boron neutron capture therapy (BNCT)[131] has been relatively ineffective in the treatment of malignant brain tumors since it has not been possible to deliver high concentrations of ^10^boron (^10^B) into individual brain tumor cells. Local chemotherapy delivery methodologies such as convection-enhanced delivery (CED)[132,133] only deliver high concentrations of ^10^B within a few millimeters of the delivery site[134]. Intravenously administered imageable dendrimers within the 7 nm to 10 nm size range bearing polyhedral borane cages[135] could be used to deliver therapeutic concentrations of ^10^B to individual brain tumor cells. This is has not been possible to accomplish with: (1) the boronated G4 dendrimer-epidermal growth factor (BD-EGF) particle, as this particle has a molecular weight of approximately 35 kDa[136], which would be consistent with a short blood half-life, and (2) the boronated monoclonal antibody[137], as the size of this antibody is close to the 12 nm physiological upper limit of pore size and the particle shape is non-spherical[108]. Spherical nanoparticles within the 7 nm to 10 nm size range bearing polyhedral borane cages would be able to deliver effective concentrations of ^10^B to individual brain tumor cells.

The premise underlying the future, successful, clinical translation of the proposed strategy is that the BBTB of malignant brain tumor microvasculature remain somewhat porous, which will necessitate that corticosteroid and VEGF inhibitor treatments be held to a minimum prior to and during the application of the proposed strategy, as it is known that these treatments significantly decrease the porosity of the BBTB. In summary, spherical nanoparticles ranging between 7 nm and 10 nm in diameter maintain peak blood concentrations for several hours and are sufficiently smaller than the 12 nm physiologic upper limit of pore size in the BBTB to accumulate to therapeutic concentrations within individual brain tumor cells. Therefore, nanoparticles bearing chemotherapy that are within this 7 to 10 nm size range can be used to deliver therapeutic concentrations of small molecule chemotherapy drugs across the BBTB into individual brain tumor cells.

## Competing interests

The author declares that they have no competing interests.

## Authors' contributions

HS conceptualized the work and wrote the manuscript.
